# Treating benign paroxysmal positional vertigo of the lateral semicircular canal with a shortened forced position

**DOI:** 10.3389/fneur.2023.1153491

**Published:** 2023-04-06

**Authors:** Beatrice Giannoni, Rudi Pecci, Federica Pollastri, Sebastiano Mininni, Giuseppe Licci, Rossana Santimone, Fabio Di Giustino, Marco Mandalà

**Affiliations:** ^1^Department of Neuroscience, Psychology, Drug's Area and Child's Health, University of Florence, Florence, Italy; ^2^Unit of Audiology, Careggi University Hospital, Florence, Italy; ^3^UOC Otolaryngology, A. Perrino Hospital, Brindisi, Italy; ^4^Department of Otolaryngology, Careggi University Hospital, Florence, Italy; ^5^Department of Otolaryngology, Azienda Ospedaliera Universitaria Senese, Siena, Italy

**Keywords:** forced prolonged position, forced position, benign paroxysmal positional vertigo, lateral semicircular canal, canalithiasis, cupulolithisias, physical therapy

## Abstract

Benign paroxysmal positional vertigo (BPPV) is the peripheral vestibular disorder that is most frequently encountered in routine neuro-otological practice. Among the three semicircular canals, the lateral semicircular canal (LSC) is the second most frequently interested in the pathological process. In most cases, LSC BPPV is attributable to a canalithiasis or cupulolithiasis mechanism. The clinical picture of LSC BPPV is that of positional nystagmus and vertigo evoked by turning the head from the supine to the side lateral position. With such a movement, a horizontal positional (and often also paroxysmal) direction-changing nystagmus is generated. Depending on whether the pathogenetic mechanism is that of canalithiasis or cupulolithiasis and depending on where the dense particles are located, LSC BPPV direction-changing positional nystagmus is geotropic or apogeotropic on both lateral sides. Due to its mechanical nature, BPPV is effectively treated by means of physical therapy. In the case of a LSC BPPV, one of the most effective therapies is the forced prolonged position (FPP), in which the patient is invited to lie for 12 h on the lateral side on which vertigo and nystagmus are less intense, to move the canaliths out from the canal (or to shift them inside of the canal from one tract to another) exploiting the force of gravity. Despite its efficacy, FPP is not always well tolerated by every patient, and it cannot be done during the diagnostic session because of its duration. The present study aimed to verify the efficacy of a different forced position, shortened forced position (SFP), with respect to the original FPP. SFP treatment would allow patients to more easily bear the forced position and physicians to control the outcome almost immediately, possibly enabling them to dismiss patients without vertigo. After 1 h of lying on the side where vertigo and nystagmus are the less intense, 38 out of 53 (71.7%) patients treated with SFP were either healed or improved. Although the outcomes are not as satisfying as those of the original FPP, SFP should be considered as a therapeutic prospect, especially by those physicians who work in collaboration with emergency departments or otherwise encounter acute patients to cure them of vertigo as soon as possible.

## Introduction

Benign paroxysmal positional vertigo (BPPV) is the peripheral vestibular disorder that is most frequently diagnosed. Patients usually complain of strong and abrupt positional vertigo elicited by vertical or horizontal movements of the head or body. The main pathogenetic mechanism is that of labyrinth lithiasis in which otoconial debris detached from the utricular macula penetrates the lumen of the canal or becomes embedded in the matrix of their cupulae (canalolithiasis and cupulolithiasis, respectively) (1, 2). In both cases, the cupulae of the semicircular canals become abnormally sensitive to gravity during head or body movements. In 5–22% of active cases referred for BPPV, the pathogenetic mechanisms alter the function of the lateral semicircular canal (LSC) (3).

Lateral semicircular canal BPPV manifests as severe rotational vertigo, occurring especially when the patient turns in bed from supine to both lateral sides. It can manifest as either of the two variants depending on the type of direction-changing positional nystagmus observed with the Head Roll test (HRT): the geotropic variant, in which such a positioning elicits a horizontal direction-changing, paroxysmal, positional nystagmus directed toward gravity on both side lateral positions, and the apogeotropic variant, in which the positioning test gives rise to horizontal direction-changing nystagmus, only rarely completely paroxysmal and directed against the gravitational vector.

The geotropic variant is caused by the canalolithiasis mechanism: the otoconial mass, initially located in the non-ampullary arm of the LSC, moves back and forth with lateral head positioning, its movement generating an ampullopetal endolymphatic flow and, thus, an excitatory discharge of the ampullary nerve by rotating toward the affected side (4, 5). Therefore, facing geotropic LSC BPPV and based on Ewald's second law, the affected side is identified by the stronger direction-changing positional nystagmus (and vertigo).

Conversely, the apogeotropic variant of LSC BPPV can be generated by either the cupulolithiasis or canalolithiasis mechanism (4, 5). In both cases, head rotation toward the healthy side provokes an utriculopetal deflection of the cupula of the affected canal that gives rise to excitatory nystagmus. Therefore, in apogeotropic LSC BPPV, due to Ewald's second law, the affected side is identified by the weaker positional nystagmus and vertigo (4–7).

Because of its mechanical origin, the LSC BPPV therapy is also mainly physical, and some liberatory maneuvers or therapeutic liberating positions are successfully used.

The efficacy of physical treatments used for LSC BPPV depends on identifying which side is affected. Even if side diagnosis is not always definite based on the single HRT, the intensities of nystagmus and vertigo are still considered the most reliable markers (3). Nevertheless, in recent years, other diagnostic tests have pinpointed some “secondary markers” that can aid in recognizing the affected side, both for LSC BPPV geotropic and apogeotropic variants (8–12). Among secondary side markers, the direction of the sitting-to-supine positioning nystagmus (so-called “lying-down nystagmus”), when appreciable, seems to be the most appropriate (10). The latter nystagmus beats toward the healthy and the affected sides in the case of the geotropic and apogeotropic variants, respectively (3, 10).

**Figure 1:**
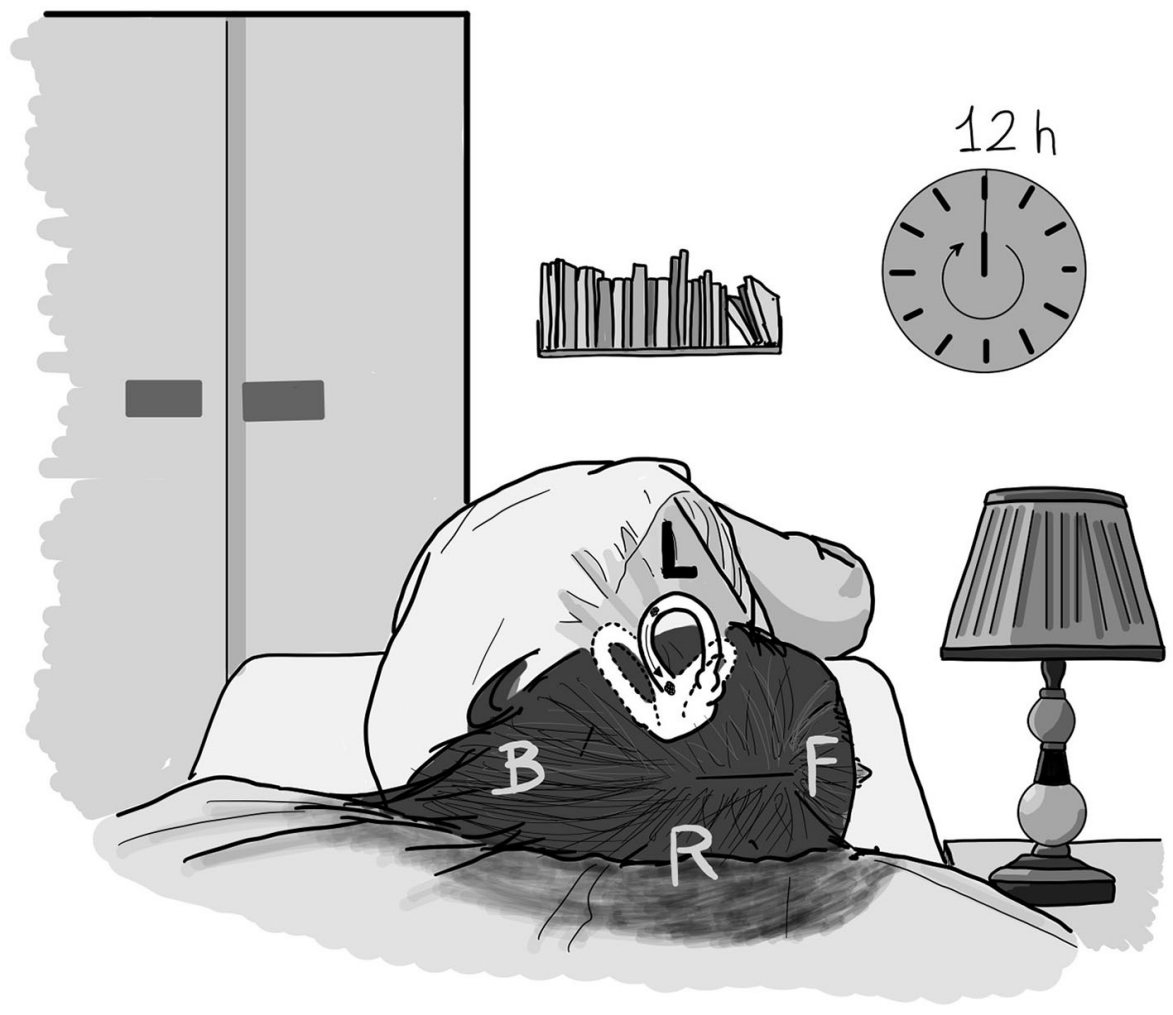
The forced prolonged position (FPP) in case of a left geotropic LSC BPPV. The patient lay for 12 h in the right-side lateral position when supine. The debris, initially located in the posterior arm of the left LSC, gradually shifted toward the vestibule under the effect of gravity. F, front; B, back; L, left; R, right.

In 1993, Baloh et al. (5, 13), first, and Lempert (14), 1 year later, achieved therapeutic success in curing geotropic LSC BPPV by performing the so-called “barbecue rotations.” These maneuvers, which aim to reposition the otoconial clot into the vestibule by turning the patient's body around the yaw axis toward the healthy side, are laborious, especially in treating overweight or elderly patients with a physical impediment. In 1997, Vannucchi et al. (15) proposed a different kind of physical therapy, which has been recently validated (16), based on the idea of obtaining a gradual leakage of the heavy particles filling the LSC by forcing the patient to lie for 12 h on the healthy side (Figure 1). The forced prolonged position (FPP) has a remarkably high success rate, and it is applicable for both LSC BPPV geotropic and apogeotropic variants,—the only difference being that, in case of an apogeotropic form, the patient should lie on the affected side. In the latter case, “reversed” FPP should act to convert the apogeotropic into geotropic LSC BBPV by dislodging the otoconial mass from the ampullary end toward the posterior arm of the canal. Once the nystagmus picture has been changed into that of the geotropic form, a second “traditional” FPP can be suggested to the patient to free the canal of the particles.

FPP is not always well tolerated by patients who are often not even sure how to correctly maintain the forced position. FPP also has the limitation of not being an immediately releasing therapy, meaning patients are compelled to go home with ongoing vertigo and have to wait for at least 24 h to check if symptoms have ceased. Moreover, due to outpatient logistics, it is not always easy to control patients the day after the therapy.

In 1998, Gufoni et al. (17) proposed another kind of physical therapy that should act by forcing the emission of the otoconial clot from the LSC lumen, taking advantage of a brisk deceleration imposed on the head. Gufoni's technique can be applied both for geotropic and apogeotropic forms and provides very good outcomes (18). Nevertheless, Gufoni's maneuver can have the same limitations as those of Semont's technique for posterior semicircular canal BPPV since patients may not be able to make such brisk movements correctly or may be afraid to perform them, thus altering the maneuver's potential outcome. Moreover, such a brisk maneuver may be difficult to apply or contraindicated in patients with physical limitations such as obesity or orthopedic problems relating to the shoulder joint, spine, or rib cage. On the contrary, the effectiveness of Gufoni's maneuver can be verified immediately after it, although to strengthen its efficacy, it is often appropriate to add FPP.

Despite the high success rate of the FPP, as originally described, patients to whom this therapy is prescribed often complain about the effort required to maintain the side lateral position for such a prolonged period; patients often feel unsure of how not to rotate while lying or sleeping and physicians cannot always verify the recovery of signs within 24 h. Because of these reasons, amongst others, we felt the need to test a kind of physical therapy that does not generate such difficulties for patients and their physicians and that could theoretically maintain the same rate of success as FPP.

We believed it necessary to “shorten” the duration of FPP so that treatment could be less stressful for patients and easier for physicians to control. Instead of 12 h, patients who were mostly manifesting a nystagmus picture attributable to a canalolithiasis mechanism were suggested to maintain the forced position for only 1 h (Shortened Forced Position [SFP]), and they would then be checked again at the end of the hour, during the same session. The rationale behind such a shortened treatment does not differ from that of the original FPP: if its outcomes should vary, it could be imputable only to a time factor and not to other variables.

## Materials and methods

From June 2019 to September 2020 at the Audiology Unit, University of Florence; Neuro-Otology Unit, University of Siena; and the Azienda Ospedaliera A Manzoni Hospital, Lecco, Italy, 53 outpatients were selected for the study group; they were selected because of positional vertigo and were diagnosed as having LSC BPPV. Among the 53 patients, 20 were men and 33 were women, with an age range of 19–81 years and an average age of 50 years. All patients underwent a standard neuro-otological examination. Vestibular spontaneous–positional nystagmus was checked in five positions (sitting, supine, right side, left side, and head-hanging), performing an HRT and Dix–Hallpike's positioning tests, using infrared video–oculoscopy. Gaze-evoked nystagmus was also checked in the same observation conditions. Ocular motility was tested at the bedside, looking at horizontal and vertical saccades and smooth pursuit eye movements. When necessary, caloric-induced nystagmus was investigated using Fitzgerald–Hallpike's technique. Patients included in the series manifested the typical clinical picture of a horizontal geotropic or apogeotropic direction-changing nystagmus, during HRT, suggesting LSC BPPV. Subjects manifesting further signs, possibly indicating other vestibular pathologies, were excluded from the study.

The involved side was determined on the basis of the HRT nystagmus intensity. The direction of the sitting-to-supine nystagmus (lying-down nystagmus) was only used as an alternative in cases with similar intensity of HRT-induced nystagmus, making the lateralization difficult.

Patients showing “atypical” nystagmus, that is, nystagmus developing without latency or a clear paroxysmal trend, evoked by different positions or positionings, without an associated sensation of vertigo or suggesting the involvement of other semicircular canals and, overall, suggesting a central vestibular system pathology, were excluded from the survey.

**Figure 2:**
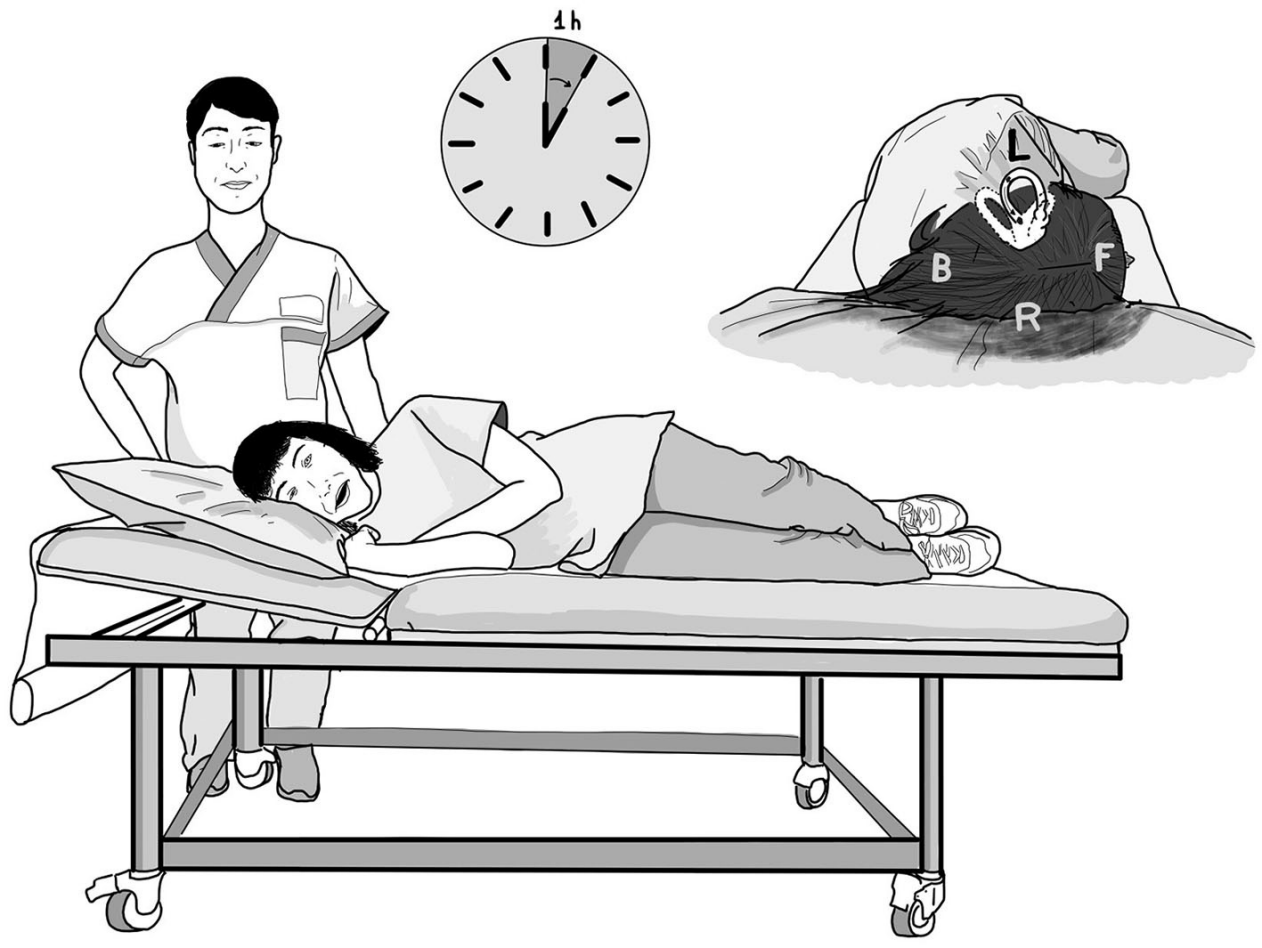
The shortened forced position (SFP) in case of a left geotropic LSC BPPV. The patient lay for 1 h in the right-side lateral position when supine. The therapy was performed in a medical environment under the supervision of a nurse. The debris, initially located in the posterior arm of the left LSC, gradually shifted toward the vestibule under the effects of gravity. F, front; B, back; L, left; R, right.

Once LSC BPPV and the corresponding affected side had been diagnosed, patients were invited to lie down on a gurney for a period of only 1 h (Shortened Forced Position [SFP]), turned onto the healthy or pathological side depending on whether they were affected by a geotropic or an apogeotropic LSC BPPV, respectively (Figure 2).

During the lying hour, patients were monitored by a nurse ascertaining that they correctly maintained the position for the whole hour without turning or getting up. The nurse also checked that the patient was able to easily tolerate the therapy and was ready to provide advice on maintaining the position or allow the patient to interrupt it in case of discomfort. After the 1-h lying period, patients were advised to carefully get up, and they were immediately re-examined; spontaneous–positional/positioning nystagmus was checked again, and the HRT and Dix–Hallpike's positioning tests were repeated, to exclude the conversion of LSC into BPPV of another canal.

During the immediate check-up, if the patients were found free of signs, they were advised to lead a normal life until they came back for a control visit within the following 72 h. As a result of the SFP, if an apogeotropic LSC BPPV was transformed into a geotropic one, patients were instructed to lie down on the opposite (healthy) side for another hour to definitively cure their pathology. If SFP provoked a canal switch, physical therapy suited to the involvement of the specific canal was applied. If LSC BPPV nystagmus was still present after SFP, qualitatively unvaried, patients were submitted to another type of therapy suitable for LSC BPPV during the same session (usually Gufoni's maneuver), adding the suggestion to perform conventional FPP at home.

The first control group comprised 35 age- and sex-matched patients who had been diagnosed in recent years as having LSC BPPV with the same clinical characteristics as the survey and treated with the original FPP. Patients belonging to such a group performed the FPP at home during the night after the diagnostic session. Within 72 h of the first evaluation, even the control group subjects received a control visit, during which the absence or presence and the typology of positional direction-changing nystagmus or other findings were checked.

Another control group was made up of 15 patients, which was demographically and clinically similar to the study group, who underwent no treatment. Patients belonging to the latter group were mostly referred to our attention by the emergency department in the hyperacute phase. Because of the intensity of their symptoms, especially after submitting to diagnostic tests, they preferred not to be physically treated but to receive symptomatic drugs. They preferred to come back for the control visit within the same time as the other cohort, out of the critical phase, to be treated with physical therapy if vertigo persisted. During the days and the nights between the diagnosis and the follow-up visits, patients were left to behave and sleep as they felt appropriate.

Patients of all three groups were checked again 1 month after healing to exclude early relapses.

The Fisher's exact test and the chi-squared test were performed on the 2 × 2 contingency tables, comparing the SFP group with each of the control groups and the two control groups with each other, respectively. The statistical analyses were performed with SPSS software version 27.0 (IBM SPSS Statistics for Macintosh, IBM Corp., Armonk, NY, USA). All statistical tests were two-sided, and a *p*-value of <0.05 was considered statistically significant.

## Results

Among the 53 patients treated with the SFP, 50 had a geotropic LSC BPPV and only 3 had an apogeotropic LSC BPPV. Right and left geotropic LSC BPPV were diagnosed in 35 and 15 cases, respectively. Apogeotropic LSC BPPV was initially diagnosed as involving the right canal in two patients and the left in the remaining cases. All patients completed SFP as indicated, without denouncing annoyance. During the period of bed rest, patients did not ask to get up or attempt to move from the suggested position. The nurse who was monitoring the performance of the treatment did not intervene/interrupt or change the patient's position.

All patients in the first control group (*n* = 35, treated with FPP) had a geotropic LSC BPPV. The right and left LSC were involved in 23 and 12 cases, respectively.

At the time of discharge, all the patients not treated (*n* = 15, second control group) showed a geotropic LSC BPPV, even though two of them had initially shown an apogeotropic LSC BPPV which, had converted to geotropic form simply by means of the diagnostic maneuvers. Among the untreated 15 study subjects, 11 had a right and 4 had a left LSC BPPV.

The SFP was entirely successful, the immediately following vestibular examination showing the absence of any sign, in 33 (66%) of 50 patients presenting with the clinical picture of a geotropic LSC BPPV. In 3 of these 50 patients (6%) of the study cohort, SFP was partially effective, being the immediate check-up indicative of a residual LSC canalolithiasis, evidenced by the permanence of a geotropic direction-changing horizontal nystagmus on the side lateral positions, although with a lesser amplitude and angular velocity than that highlighted at the time of diagnosis. Compared to the onset, in these three subjects, positional vertigo was also much reduced, both in intensity and duration. Therefore, the SFP had an overall positive result in 36 out of 50 (72%) patients with a geotropic LSC BPPV.

Moreover, SFP was effective in two-thirds of patients presenting with an apogeotropic LSC BPPV; essentially, at the control visit, in these two subjects, LSC BPPV positional nystagmus was absent in one subject and changed into the geotropic type in the other subject. The subject for which the positional nystagmus converted from apogeotropic into geotropic was successfully treated later by performing SFP on the healthy side, during the same session. Conversely, SFP proved unsuccessful in the remaining subject affected by an apogeotropic LSC BPPV.

Consequently, considering the results obtained for geotropic and apogeotropic forms together, it can be stated that SFP was completely or partially effective in 38 out of 53 (71.7%) patients.

After SFP, in 13 out of 50 (26.0%) patients with geotropic LSC BPPV, positional nystagmus and vertigo were still evident and completely unvaried in their qualitative and quantitative characteristics. By adding to the latter subjects the patient with the apogeotropic form who was neither negative nor improved after the SFP, it emerged that the SFP was ineffective in 14 out of 53 (26.4%) of the study cohort subjects.

Curiously, 1 among the 50 patients (2.0%) who showed a geotropic LSC BPPV before SFP manifested the clinical picture of an apogeotropic LSC BPPV after 1 h of lying down on the hypothetical healthy side. The case of this patient must also be considered when calculating those for which the therapy was not effective, with the relative percentage raising to 28.3%.

After therapy, in the study group, none of the geotropic or apogeotropic LSC BPPV cases were transformed into a BPPV due to the involvement of other semicircular canals (Table 1).

**Table 1:** Distribution of patients of the study group with regard to LSC BPPV variant and SFP outcome.

**Patients (study group)**	**LSC BPPV variant**	**Healed or improved**	**Transformed**	**Unvaried**
50	Geotropic	36 (72%)	1 (2%)	13 (26%)
3	Apogeotropic	1 (33.3%)	1 (33.3%)	1 (33.3%)

None of the subjects cured with SFP manifested a recurrence nearby.

The first control group comprised 35 age- and sex-matched patients treated for geotropic LSC BPPV that served as a cohort in a previous study on the efficacy of traditional FPP for LSC BPPV (15). After having maintained the FPP, the therapy could be considered effective in 32 of 35 (91.4%) subjects. Essentially, 26 of 35 patients (74.3%) were completely cured, showing no signs, and 6 patients (17.1%) manifested a canal switch into the ipsilateral posterior semicircular canal (subsequently treated and cured with suitable physical therapy). From a clinical point of view, only three patients (8.6%) did not show any changes at the first review; they took longer to recover (15, 40, and 43 days). During these recovery periods, the patients repeated the therapy at home and underwent cranial magnetic resonance imaging (MRI) that excluded a possible central cause. In the end, recovery was complete in all cases.

After the therapy, none of the patients belonging to the FPP group showed a “paradoxical” transformation of a geotropic LSC BPPV into an apogeotropic LSC BPPV. Even those patients who were cured following FPP did not show short-term relapses.

A total of 4 out of 15 patients (26.7%) in the second control group who were not treated with any physical therapy came to the follow-up visit without manifesting any symptoms or signs. The remaining 11 (73.3%) subjects still had an unvaried clinical picture.

On comparing the SFP and FPP groups with the second control group (patients who were not treated with any physical therapy), we observed that there was a statistically significant difference in the frequencies of a positive outcome, with a *p*-value for both the Fisher's exact test and the chi-squared test of 0.002 and < 0.001, respectively, for the analysis of the SFP group and the FPP group.

Comparing the SFP and FPP groups also showed significant differences in positive outcome; with the *p*-value equal to 0.031 on Fisher's exact test and equal to 0.025 on the chi-squared test. This indicates that traditional FPP was statistically more effective than SFP (Table 2).

**Table 2:** Contingency table with the three groups of patients and treatment outcome.

**Group**	**Positive outcome *n* (%)**	**Negative outcome *n* (%)**	**Total**
*SFP*	38 (71.7)	15 (28.3)	53
*FPP*	32 (91.4)	3 (8.6)	35
*NT*	4 (26.7)	11 (73.3)	15
Total	71 (71.0)	29 (29.0)	100

Chi-squared test: SFP-NT, p = 0.002; FPP-NT, p < 0.00; SFP-FPP, p = 0.025.

Fisher's exact test: SFP-NT, p = 0.002; FPP-NT, p < 0.001; SFP-FPP, p = 0.031.

SFP, shortened forced position; FPP, forced prolonged position; NT, not physically treated.

## Discussion

Encountering patients affected by LSC BPPV is relatively habitual in dizziness clinics. Clinical diagnosis is often easy for expert physicians based on the finding of a horizontal direction-changing paroxysmal positional nystagmus onto side lateral positions, which is directed toward the ground in the case of the geotropic form and opposite to the gravity vector in the case of the apogeotropic variant. The identification of the affected side is also relatively simple, especially in the geotropic variant, considering the intensities of nystagmus and vertigo on both side lateral positions and the directions of the fast phase of the sitting-to-supine positioning nystagmus that patients often show as an additional lateralizing sign (10). Other side markers exist, but they all have lower sensitivity and specificity (8–12). Given the side dependence of the most-effective physical treatments, locating the affected side is crucial in establishing the correct therapeutic approach.

Among the currently available and validated physical treatments, the FPP, originally described by Vannucchi et al. (15), has a high success rate in curing at least geotropic LSC BPPV. The FPP involved instructing the patient to lie down on the healthy side for 12 h, with the affected ear facing upward. In such a position, the posterior arm of the affected LSC was oriented vertically in such a way that the otoconial cluster inside it could gradually shift toward the utricle, under the effects of gravity (Figure 1). Because of the friction between the canal walls and the clot in the endolymph, the debris was believed to drag slowly. For this reason, FPP was believed to take quite a long time to be effective, and therefore should not be performed, as was the case for other maneuvers suitable for BPPV in other canals, during the same outpatient visit in which the diagnosis was made. Hence, once the diagnosis was reached, patients were asked to go home and carry out FPP on their bed or sofa. Patients were also given advice and indications on how to avoid turning around during the 12 h of positioning (i.e., arranging a pillow barrier behind the back) and how to behave in case of needing to get up during such a long period (avoiding abrupt forward and backward head movements). Nevertheless, the proper execution of the therapy could not be controlled neither could patients be protected from the possibility of sudden dizziness caused by inappropriate movements. It is not possible to establish with certainty whether the FPP was appropriately performed in terms of times and methods because patients were alone at home and could not control themselves, as they were often asleep. Furthermore, patients who performed FPP often complained of difficulty and annoyance in maintaining the obligatory position due to pain in the back and shoulder or numbness in the limbs. In addition, physicians could not evaluate the treatment outcomes before a 24-h period. Due to the presumed therapeutic mechanism, which is the gradual setting of debris out of the canal, physical therapies such as FPP were believed to be more efficient in the case of canalolithiasis rather than in that of cupulolithiasis, even if the risk of an abrupt detachment of the clot embedded in the cupula or located near it (within the short arm of the canal) cannot be excluded. As mentioned above, despite the high percentage of resolution rate of this kind of physical treatment, patients often reported some drawbacks, such as low tolerance of such prolonged bed rest, potentially inducing them to interrupt the correct position or leave it before it was time. For the above reasons, when facing patients affected by LSC BPPV, we considered testing the efficacy of the FFP technique by using the same pathophysiological principle but shortening the resting period on the lateral side to just 1 h (SFP) in order to reduce subject discomfort, apply the treatment to a larger number of patients, perform therapy during the diagnostic session, control its correct execution, and immediately verify its outcome.

In addition, we believed that a shortened treatment could be a useful tool for physicians dealing with acute patients and/or as a first-line therapy before specialist examination in a first aid context.

The application of SFP has been globally successful. Essentially, considering a combination of patients who were cured, or had a positive outcome toward being cured, and those whose symptoms and signs were reduced after the shortened approach, a success rate of 71.7% was reached. This consistent positive result should always encourage patients to at least attempt this type of therapy whenever possible and whenever allowed by the logistics of the healthcare environment in which the diagnosis is made.

Despite such a high success rate, SFP was still statistically less effective than FPP. Therefore, SFP cannot be considered a substitute for the FPP. Rather, although the shortened therapy does not have exactly the same therapeutic potential as the FPP, it should still be attempted, at least for screening purposes, to reduce the number of patients to whom the more arduous FPP should be applied. Even if the SFP proves ineffective at the time of the first diagnostic evaluation, the FPP can be done at home, having had the opportunity to directly show patients how the therapy should be performed and, thus, better inform and reassure them.

On the contrary, using both SFP and FPP makes achieving a cure or an improvement of the disease statistically much more likely than not treating patients or administering them with symptomatic drugs alone.

Most of the patients in the study group had an LSC BPPV in the geotropic form. This feature makes the comparison with the control groups even more homogeneous. In fact, both the first and the second control groups consisted entirely of patients with LSC canalolithiasis with a geotropic direction-changing nystagmus on the side lateral positions. Regarding the first control group, cases had been deliberately selected with the nystagmus picture of a geotropic LSC BPPV, which is, thus, certainly attributable to the canalolithiasis mechanism. In the case of the second control group, which comprised untreated patients, the absolute prevalence of geotropic forms was probably due, in addition to chance, to the fact that most of our patients came from the emergency room or hospital wards in which we were called to provide our advice since the onset of symptoms.

However, it is inherent in the rationale of the FPP, and overall, in that of therapeutic techniques based on decanting, that the likelihood of effective treatment is higher when the otoconial debris is free to move in the lumen of the canal rather than when it is incorporated in the gelatinous matrix of the cupula or located into the ampullary dilatation.

The SFP was also effective in two patients presenting with an LSC BPPV in the apogeotropic form; however, the small size of this sample does not allow for any statistically valid considerations. The reason why the SFP was found to be curative, even for apogeotropic forms, could be that, in such cases, despite being located in the periampullary region, the otoconial debris is free to move into the canal lumen, under the effect of gravity.

Nevertheless, as things stand, all the considerations made on the efficacy of the SFP, as well as those made for the FPP, must be considered relevant for geotropic LSC BPPV and, for this reason, for canalolithiasis only.

The SFP was found to be applicable to all patients; none of them was unable to complete it and no one complained of any significant disturbances during its execution.

All subjects undergoing SFP were checked immediately after 1 h bed of rest and within 72 h after therapy; no other instructions about movements to avoid or positions to hold were given during the period before the check-up. At control, none of the patients cured following SFP showed a recurrence of the initial symptoms and signs. Moreover, none of the patients had a short-time relapse within a month of the initial diagnosis.

The above observation leads to the consideration that, when reached, the favorable outcome of SFP, similar to that of FPP, is stable. Being two therapies based upon the same rationale and the same pathophysiology, the different outcomes obtained with FPP and SFP can be attributed only to time. In the remaining 13 out of 50 patients with geotropic LSC BPPV treated with SFP, an insufficient period of resting must be the explanation for the therapeutic failure. However, resting time is crucial to a patient's tolerance to this physical treatment. Even the finding that, in the SFP group, unlike that of traditional FFP, there were some cases of partial improvement of symptoms and signs, meaning patients were not completely cured after 1 h of resting only, could be an indication that the time factor is crucial.

Among patients with geotropic LSC BPPV treated with SFP, we observed one case in which the forced position led to a conversion of the initial geotropic nystagmus pattern to that typical of an apogeotropic LSC BPPV. Such a transformation could be explained by hypothesizing that while lying on the healthy side, the patient assumed a position such that the mass of debris fell from the non-ampullary arm toward the ampullary end of the canal lumen instead of heading toward the utricle. It cannot even be completely ruled out that, in this case, we misidentified the LSC BPPV side, asking the patient to lie on the affected one, thus making the debris move toward the ampullary arm of the canal instead of toward the vestibule.

Only three patients with apogeotropic LSC BPPV were treated with SFP; among those three patients, we had the same percentage of patients cured, transformed, and unvaried. Owing to the small number of patients, these results are not significant. However, in dealing with this type of LSC BPPV, a poorer outcome of SFP (and of FPP as well) is conceivable, considering that it could be generated by the cupulolithiasis mechanism. Moreover, even in the case of canalolithiasis, due to its initial periampullary location, shifting the debris toward the posterior arm of the canal can be more difficult. There are no other documented results available in the literature regarding the treatment of the apogeotropic form either with SFP or FPP.

The mechanism by which one case of apogeotropic LSC BPPV was directly cured, only after a single hour of “reverted” SFP, without showing any phase of geotropic direction-changing positional nystagmus, could be that we were wrong in identifying the involved side. By lying (incorrectly) on the healthy side, a free clot located in the LSC short arm could have dispersed into the vestibule, or a mass adhering to the utricular face of the cupula could have been detached and fallen into the vestibule.

In the second control group, we still had a 26.7% resolution of the disorder, even without performing any physical therapy. This data could be explained by the fact that, in general, BPPV has a 20% possibility of spontaneous resolution within a month (3). However, it can also be hypothesized that once at home and lying on their backs, these patients could have assumed an obligatory position precisely on the side suitable to free the canal (which is always the one where the patient is least worse off).

However, the statistical analysis clearly demonstrated how treating patients is correlated with a better outcome.

It should be noted that LSC BPPV therapy should also be eminently physical. Although less common with respect to BPPV in other canals, LSC BPPV is a particularly incapacitating type of vertigo; sometimes symptoms are so intense that they are continuous and long-standing rather than positional.

Diagnosis should be made with a few focused maneuvers capable of framing the pathology and identifying the affected side while causing minimal discomfort to the patient; clarifying the affected side is essential in determining the performance of the suitable therapeutic approach.

Moreover, the overriding aim of physical therapy is to achieve recovery in the shortest time possible, while causing the least possible discomfort to the patient. At least in the case of a geotropic LSC BPPV, the implementation of SFP, performed during the outpatient visit, could be an exploitable therapeutic possibility to achieve almost an immediate outcome and to facilitate the discharge of patients without vertigo.

The SFP does not require any special equipment, just a gurney, a pillow, and an environment where the patient can rest under supervision. Such a shortened treatment could be applied as a first-line choice or used by physicians working with acute patients.

Even if its outcome is not as exceedingly satisfying as that of FPP, in our opinion, SFP should be attempted anyway before submitting patients to other procedures involving brisk movement, such as Gufoni's maneuver, which can be more bothersome and not applicable to everyone, or before instructing them to conduct traditional FPP at home.

Moreover, achieving the resolution of symptoms and signs in such a short time and with physical therapy alone, especially in a first aid context, can also help in the differential diagnosis of vertigo of possible central origin.

A limitation of the present study is that we did not document the overall degree of acceptability of the different therapeutic approaches. It could certainly be useful in future studies, even larger ones, to administer a questionnaire that investigates this point in order to establish how exactly SFP is more tolerable than FPP and no treatment.

In addition, it should be noted that data on the efficacy of the three different therapeutic approaches are to be reported to groups of patients that were evaluated in different periods.

## Data availability statement

The raw data supporting the conclusions of this article will be made available by the authors, without undue reservation.

## Ethics statement

All procedures performed in studies involving human participants were in accordance with the ethical standards of the institutional and/or national research committee and with the 1964 Helsinki Declaration and its later amendments or comparable ethical standards. Informed consent was obtained from all individual participants included in the study.

## Author contributions

BG wrote the paper, contributed in conceiving the study, collected and studied patients, and provided discussion and interpretation of results. RP contributed in writing the paper, collecting and studying patients, and providing discussion and interpretation of results. FP contributed in writing the paper, collecting and studying patients, and interpreting clinical and statistical data. SM contributed in writing the paper and collecting and studying patients. GL performed statistical analysis and contributed in interpreting results. RS and FD contributed in recruiting patients. All authors contributed to the article and approved the submitted version.
